# Cross-Cultural Adaptation and Initial Psychometric Evaluation of the Japanese Version of the Chronic Kidney Disease Self-Efficacy Scale (CKDSE-J)

**DOI:** 10.3390/healthcare14203393

**Published:** 2026-10-12

**Authors:** Tingting Geng, Yuki Kajiwara, Michiko Morimoto

**Affiliations:** 1 Doctor’s Program, Graduate School of Health Sciences, Okayama University, Okayama 700-8558, Japan; p9oy1quj@s.okayama-u.ac.jp; 2 Faculty of Health Sciences, Okayama University, Okayama 700-8558, Japan; mmorimoto@cc.okayama-u.ac.jp

**Keywords:** chronic kidney disease, self-efficacy, self-care, cultural adaptation, psychometric evaluation, exploratory structural equation modeling

## Abstract

**Highlights:**

**What are the main findings?**

- The 23-item CKDSE-J showed improved overall model fit with four-factor Target ESEM compared with restrictive CFA, although several autonomy items showed weak target loadings and cross-loadings.
- Proposed CKDSE-J subscale scores were positively associated with self-care behavior, with acceptable internal consistency across the four proposed subscales.

**What are the implications of the main findings?**

- The CKDSE-J may provide a CKD-specific approach to assessing multiple aspects of self-efficacy related to CKD management among adults with CKD stages G3a–G5 not receiving dialysis.
- Independent replication and further psychometric evaluation are needed before the revised 23-item structure, particularly the autonomy factor, can be considered established.

**Abstract:**

**Background/Objectives**: Self-efficacy is an important determinant of self-care among adults with chronic kidney disease (CKD). The Chronic Kidney Disease Self-Efficacy Scale (CKD-SE) is a disease-specific measure, but its use has not been reported in Japan. This study translated and culturally adapted the CKD-SE into Japanese and conducted an initial psychometric evaluation. **Methods**: A cross-sectional survey was conducted among Japanese adults with CKD stages G3a–G5 not receiving dialysis. Structural validity and internal consistency were evaluated in 227 participants, and construct validity was evaluated in 222. Structural validity was examined using confirmatory factor analysis (CFA) and exploratory structural equation modeling (ESEM). **Results**: The original 25-item four-factor CFA showed a poor fit, and ESEM yielded an improper solution involving Item 19. Sequential evaluation resulted in a 23-item structure. The 23-item CFA showed mixed fit, whereas Target ESEM showed improved overall fit (CFI = 0.935, TLI = 0.902, RMSEA = 0.060, SRMR = 0.033). However, several autonomy items showed weak target loadings and cross-loadings. Proposed subscale scores correlated positively with self-care behavior (r = 0.27–0.46), and McDonald’s omega ranged from 0.84 to 0.90. **Conclusions**: The 23-item CKDSE-J showed improved overall model fit under Target ESEM, but the structural definition of autonomy remained uncertain. Because the structure was refined and evaluated in the same sample, independent replication and further psychometric evaluation are needed.

## 1. Introduction

Chronic kidney disease (CKD) is a progressive condition that, without effective management, can lead to cardiovascular events and end-stage renal disease requiring renal replacement therapy such as dialysis. Slowing its progression is therefore a major clinical priority. Clinical guidelines recommend that preventing CKD progression requires sustained self-care behaviors, including dietary management, medication adherence, blood pressure control, physical activity, and smoking cessation [1]. Because these behaviors must be incorporated into daily life over the long term, psychological factors that support the initiation and maintenance of self-care behavior are clinically important.

Recent research has increasingly examined self-management support for adults with non-dialysis CKD, including educational, behavioral, multidisciplinary, and population health approaches, to facilitate treatment adherence, lifestyle modification, and daily disease management [2,3,4]. Within this body of work, self-efficacy has been identified as a modifiable determinant of self-care behavior in adults with CKD [5]. Interventions aimed at enhancing patient activation and self-management have been applied in CKD, with meta-analytic evidence indicating improvements in self-efficacy and self-management-related outcomes [6]. However, heterogeneity in intervention effects has also been reported [6]. Differences in measurement instruments and conceptual frameworks may plausibly contribute to heterogeneity in observed intervention effects. The Consensus-based Standards for the selection of health Measurement INstruments (COSMIN) provide an internationally established methodological framework for evaluating the measurement properties of health-related measurement instruments and emphasize the importance of selecting instruments that are appropriate for the construct and context of use [7,8,9]. Consistent with this, a review of self-efficacy instruments used in CKD populations found that available measures range from generic chronic-disease scales to CKD-specific instruments and recommended that researchers select tools according to the specific population and purpose of assessment [10]. Bandura [11,12] emphasized that self-efficacy should be assessed in relation to specific domains of functioning rather than as a global disposition. Accordingly, a CKD-specific instrument may provide better alignment when evaluating self-efficacy related to CKD management. Although generic self-efficacy instruments are useful for broad comparisons across chronic diseases, they may not adequately capture confidence in CKD-specific self-management tasks.

The Chronic Kidney Disease Self-Efficacy Scale (CKD-SE) was developed to assess disease-related self-efficacy in performing CKD self-care behaviors [13]. The scale was designed in the context of CKD progression prevention and evaluates confidence across four factors—autonomy, self-integration, problem-solving, and seeking social support—providing a framework for assessing multiple aspects of adults’ perceived capability to engage in CKD management. The CKD-SE was subsequently translated into Indonesian and used among adults with early-stage CKD, with the Indonesian version demonstrating satisfactory content validity and internal consistency [14]. However, because the CKD-SE was originally developed for Taiwanese adults with early-stage CKD (G2–G3), its applicability to adults with more advanced CKD stages, who face greater self-care demands, remains unclear. Moreover, consistent with established principles of cross-cultural measurement adaptation [15], a translated instrument’s measurement properties cannot be assumed to hold in a new linguistic and cultural context without empirical evaluation, and the psychometric properties of the CKD-SE have not yet been evaluated in Japan.

In Japan, although new dialysis introductions have recently declined [16], preventing progression to kidney failure remains an important goal of self-care support for adults with non-dialysis CKD. However, self-efficacy assessment in this population has generally relied on generic instruments rather than CKD-specific ones, with previous work often focused on diabetic nephropathy rather than CKD more broadly [17,18]. Despite continuing interest in self-efficacy as a component of CKD self-management, a Japanese CKD-specific measure of self-efficacy with established psychometric properties remains lacking. Demonstrating that a CKD-specific measure can be usefully applied in this context may therefore carry both clinical value—by helping clinicians evaluate multiple aspects of adults’ CKD-related self-efficacy and inform more individualized support—and research value by providing a construct-aligned outcome measure for evaluating self-care interventions. Moreover, because self-efficacy beliefs may be influenced by cultural context [19], empirical evaluation of the CKDSE-J in Japanese adults with CKD is warranted. Therefore, this study translated and culturally adapted the CKD-SE into Japanese and evaluated its structural validity, reliability, and construct validity among Japanese adults with CKD stages G3a–G5, with particular attention to whether the original four-factor structure was adequately represented in this population.

## 2. Methods

### 2.1. Study Design

This cross-sectional psychometric study was conducted from 19 June–24 October 2019 at a 1000-bed university hospital in western Japan. The hospital’s nephrology department serves a wide range of adults with CKD, including those with primary and diabetic kidney disease.

### 2.2. Participants and Sample Size

The study’s eligibility criteria included adults aged 20 years or older who had been diagnosed with CKD stages G3a–G5 (selected because these stages are associated with a heightened risk of disease progression and represent a key target population for predialysis management and self-care support interventions [20,21]). Eligibility with respect to kidney function was determined using the most recent eGFR value available at the time of recruitment, and an eGFR below 60 mL/min/1.73 m^2^ was required. According to the KDIGO classification, the eGFR ranges for CKD stages G3a–G5 are 45–59, 30–44, 15–29, and <15 mL/min/1.73 m^2^ for G3a, G3b, G4, and G5, respectively [1]. Participants were not receiving dialysis (i.e., non-dialysis-dependent CKD). CKD stage used in the analyses was classified separately using the eGFR value available at the time of the survey to account for possible changes in kidney function between recruitment and the survey. Because self-care demands may increase as CKD progresses, the aim of this study was to evaluate whether the CKDSE-J could adequately assess self-efficacy in adults with CKD stages G3a–G5. Consistent with COSMIN’s emphasis on evaluating content validity in relation to a specific target population [8,9], the study sample was therefore defined to match this target population. Participants were required to be receiving outpatient or inpatient treatment at a university hospital and provide voluntary informed consent. The exclusion criteria were prespecified in the study protocol and included individuals unable to complete the self-administered questionnaire because of severe visual or cognitive impairment; those with psychiatric disorders; those with kidney diseases for which deterioration was not considered preventable through self-care; those with severe or active comorbid conditions for which treatment other than CKD management was the predominant clinical priority; and those with reduced kidney function following renal surgery. For inpatients, those hospitalized specifically for dialysis initiation were excluded, whereas those admitted for vascular access creation or the first stage of stepwise initiation of peritoneal dialysis using the Moncrief–Popovich technique were eligible. Eligibility was assessed by the attending physicians using these prespecified criteria, available clinical information, and clinical judgment. No numerical cutoff was specified for cognitive impairment; rather, the criterion was whether the patient was able to complete the self-administered questionnaire. Similarly, no numerical threshold was prespecified for the severity or treatment burden of comorbid conditions; this exclusion criterion was applied when, in the attending physician’s clinical judgment, treatment of a comorbid condition rather than CKD management constituted the predominant clinical priority. All patients determined to be eligible were consecutively invited to participate from both outpatient and inpatient settings at the study site. Because eligibility screening was conducted by multiple attending physicians during routine clinical care and no centralized screening log was maintained, the total number of patients assessed before eligibility was established and the numbers excluded for individual criteria could not be reconstructed retrospectively.

The sample-size target was originally determined for the planned CFA based on commonly cited recommendations suggesting that samples of approximately 200 or more may be adequate under appropriate conditions [22]. We aimed to recruit at least 270 participants, assuming a 75% response rate, to ensure that the analysis included a minimum of 200 participants.

### 2.3. Translation and Cultural Adaptation Process

The Japanese version of the CKD-SE was developed according to the methodology established by the International Society for Pharmacoeconomic Research and Outcomes Task Force [15]. Permission to translate the original scale was obtained from the original developer, Professor Chiu-Chu Lin. Although the CKD-SE was originally developed in Chinese, the version provided by the original developer for the present Japanese adaptation was in English. Therefore, this developer-provided English version was used as the source version for translation into Japanese. The research team did not independently verify semantic equivalence between the Chinese and English versions.

Three independent translators—a Japanese university faculty member specializing in English education, an English instructor with study experience in the United States, and a Japanese nursing researcher with a degree and clinical experience in an English-speaking country—each separately produced Japanese versions. Discrepancies among the three versions were resolved through discussion and consensus among the research team (Y.K. and M.M., faculty co-authors with expertise in nephrology nursing and instrument translation) together with the translators. A professional translation company performed the back translation into English. The back-translated English version was reviewed by the original developer, who confirmed that there were no problems with the translated content.

A pretest with two Japanese patients with CKD, one aged <65 years and the other aged ≥75 years, served as the study’s cognitive debriefing step. Participants were asked whether the meaning of the translated items was understandable and whether any items were difficult to answer. Neither participant reported particular difficulties in understanding or responding to the items, and no wording revisions or cultural adaptations were made based on their feedback. Feedback was obtained verbally during the pretest but was not systematically documented or formally analyzed. The cognitive debriefing therefore primarily assessed comprehensibility and difficulty in responding and did not systematically assess patient perspectives on item relevance or comprehensiveness. The original CKD-SE used a 1–10 Likert response scale, which was later revised by the developers to response options ranging from 0 (no confidence) to 10 (great confidence). In accordance with the developers’ instructions, only the revised 0–10 response scale was translated and used in this study.

### 2.4. Data Collection Procedure

Potentially eligible adults were consecutively identified in outpatient and inpatient settings. Eligibility was determined by the attending physician according to the prespecified inclusion and exclusion criteria described above. All patients determined to be eligible were invited to participate. Individuals who agreed to participate received a self-administered questionnaire comprising items on demographic and clinical characteristics, the CKDSE-J, and the CKDSC-J. No directly identifying information was recorded on the questionnaire. Each participant was assigned a unique study ID. Demographic and clinical data were obtained from electronic medical records and linked to the questionnaire using the study ID. A temporary linkage key connecting participants’ names with their study IDs was permanently deleted after data linkage was completed. Outpatients completed the questionnaire at home and returned it by mail, whereas inpatients completed it during their hospital stay using a leave-behind method. Neither researchers nor nurses were present while participants completed the questionnaires or provided assistance with their responses. For outpatients, the presence of family members or other persons during questionnaire completion was not recorded. Completion required approximately 20–30 min. Outpatients were given one month to return the questionnaire by post, whereas inpatients were allowed one to two weeks, depending on their individual circumstances. The numbers of questionnaires distributed in the outpatient and inpatient settings were recorded at recruitment; however, recruitment setting was not retained at the individual level in the analytical dataset. Therefore, setting-specific responses and analysis counts could not be determined.

### 2.5. Measures

#### 2.5.1. Demographic and Clinical Characteristics

The following participant characteristics were collected: sex, age, body mass index, eGFR, CKD stage, comorbidities, CKD duration, receipt of CKD education, living situation, and employment status.

#### 2.5.2. CKD-SE Instrument (CKDSE-J)

The CKD-SE, developed by Lin et al. [13], is a disease-specific instrument designed to assess self-efficacy among adults with CKD. The scale comprises 25 items across the following four subscales: autonomy (confidence in expressing concerns and making independent decisions; eight items: 1, 4, 9, 11, 12, 17, 18, and 21), self-integration (perceived ability to incorporate CKD management into daily life; seven items: 10, 15, 16, 19, 22, 23, and 24), problem-solving (confidence in acquiring and applying disease-related knowledge; six items: 2, 3, 5, 8, 13, and 20), and seeking social support (belief in obtaining help from family, friends, and healthcare providers; four items: 6, 7, 14, and 25). The original CKD-SE demonstrated high internal consistency (Cronbach’s α = 0.94) and satisfactory test–retest reliability (r = 0.72); its construct validity was supported by EFA [13]. In the present study, the Japanese version of the CKD-SE (CKDSE-J), developed as described in Section 2.3, was used to examine its psychometric properties. Each item was rated using response options ranging from 0 (no confidence) to 10 (great confidence), with higher scores indicating greater self-efficacy. The proposed subscale allocation, response options, and scoring instructions for the 23-item CKDSE-J are provided in Supplementary Table S1.

#### 2.5.3. CKD Self-Care Scale—Japanese Version (CKDSC-J)

The Japanese version of the Chronic Kidney Disease Self-care Scale (CKDSC-J), originally developed by Wang et al. [23] and later translated and validated in Japanese by Kajiwara and Morimoto [24], was utilized to assess self-care behaviors among adults with CKD. The CKDSC-J comprises 15 items across the following five domains: medication management, dietary habits, physical activity, smoking cessation, and blood pressure management. Each item is rated on a five-point scale ranging from “never” to “always,” with higher scores indicating greater engagement in self-care behaviors. The CKDSC-J was selected as an external criterion because it assesses self-care behaviors consistent with guideline-recommended CKD management [1]. The Japanese version has demonstrated acceptable internal consistency and structural validity [24].

### 2.6. Ethical Considerations

This study was conducted in accordance with the Declaration of Helsinki and was approved by the Okayama University Graduate School of Medicine, Dentistry and Pharmaceutical Sciences and Okayama University Hospital, Ethics Committee (K1905-023). All potential participants received written and verbal explanations of the study purpose and procedures, the voluntary nature of participation, confidentiality, and the collection and linkage of medical record data with questionnaire responses. Participants were also informed that the questionnaire was anonymous and that their data would be coded and handled in a manner that would not allow individuals to be identified. The questionnaire was distributed only to individuals who agreed to participate, and completion of the questionnaire with the consent box checked was regarded as indicating informed consent.

### 2.7. Statistical Analysis

#### 2.7.1. Data Preparation and Descriptive Analysis

Data were analyzed using SPSS version 21.0 and Mplus version 7.4. Analytical samples were defined according to the data required for each analysis. Of the 244 respondents, 17 had missing CKDSE-J item responses and were excluded from analyses of the CKDSE-J, leaving 227 participants with complete CKDSE-J data. These 227 participants constituted the primary analytical sample for structural validity and internal consistency analyses. Among them, 5 had missing CKDSC-J item responses; therefore, analyses involving both the CKDSE-J and CKDSC-J were conducted using the 222 participants with complete data on both instruments. No imputation was performed. Missingness was examined by instrument and item. To describe the potential impact of missing CKDSC-J data on the construct validity sample, demographic and clinical characteristics were summarized separately for the 222 participants with complete CKDSE-J and CKDSC-J data and the five participants with complete CKDSE-J data but missing CKDSC-J data. Because the latter group was very small (*n* = 5), inferential between-group comparisons were not performed. Participant characteristics and CKDSE-J item responses were summarized using descriptive statistics, including means, standard deviations, frequencies, and percentages. Normality was assessed using skewness and kurtosis; values of skewness < |2.0| and kurtosis < |7.0| were considered to indicate an approximately normal distribution [25].

#### 2.7.2. Structural Validity Assessment

Structural validity was evaluated using robust maximum likelihood estimation (MLR). First, the hypothesized 25-item four-factor CFA was fitted, with each item loading only on its designated factor and all non-target cross-loadings fixed to zero. If the CFA showed inadequate global fit, local areas of model misfit were examined diagnostically using modification indices and associations among conceptually similar items. These diagnostics were used to assess whether the independent-cluster CFA assumption of zero cross-loadings might be overly restrictive rather than to introduce correlated residuals solely to improve model fit.

A four-factor exploratory structural equation modeling (ESEM) model with oblique GEOMIN rotation was estimated for the original 25 items to examine whether allowing cross-loadings provided a more adequate representation of the covariance structure than the restrictive independent-clusters CFA. GEOMIN rotation was used at this stage because it provides a relatively exploratory ESEM solution in which the number of factors is specified while the pattern of primary and non-primary loadings is estimated from the data [26,27]. All items were allowed to load on all four factors, and the factors were allowed to correlate. The ESEM solution was evaluated in terms of global model fit, factor interpretability, factor correlations, and the presence of improper solutions. When item-level measurement problems were identified, they were considered together with conceptual redundancy in determining whether item removal was warranted. Because the 25-item ESEM yielded an improper solution involving Item 19, an intermediate 24-item model excluding Item 19 alone was estimated using both CFA and GEOMIN ESEM to determine whether removal of Item 19 resolved the improper solution and to evaluate the remaining model misfit. Item-level diagnostics and conceptual overlap were then examined before any further item removal.

Following these sequential evaluations, the resulting 23-item structure was examined using both CFA and Target ESEM. In the Target ESEM, the original factor allocation was specified as the target structure. Non-target loadings were targeted toward zero rather than fixed exactly to zero, and oblique target rotation allowed the four factors to correlate [26,28]. This approach allowed potentially meaningful cross-loadings to be estimated while retaining the hypothesized four-factor structure. Target rotation was considered appropriate because the four-factor structure was specified a priori from the original CKD-SE, making it possible to evaluate this theoretically defined structure in a relatively confirmatory manner while still permitting small non-target cross-loadings [27,28,29]. The Mplus syntax for the 23-item four-factor Target ESEM is provided in Supplementary Text S1.

Model fit was evaluated using the chi-square statistic (χ^2^), the comparative fit index (CFI), the Tucker–Lewis index (TLI), the root mean square error of approximation (RMSEA) with its 90% confidence interval, and the standardized root mean square residual (SRMR). Fit indices were interpreted collectively rather than as rigid pass/fail criteria [30]. The Akaike information criterion (AIC) and Bayesian information criterion (BIC) were additionally reported as supplementary indices for comparisons between models fitted to the same set of items, with lower values indicating relatively better fit. Standardized target and non-target loadings, their standard errors, standardized residual variances, and factor correlations were examined to evaluate factor interpretability and model admissibility.

#### 2.7.3. Reliability, Subgroup Comparisons, and Construct Validity

Internal consistency reliability was assessed using the 227 participants with complete CKDSE-J data, with ω ≥ 0.70 considered acceptable [31]. McDonald’s omega (ω) was estimated separately for each hypothesized subscale based on the prespecified item allocation using the omega() function in the psych package in R version 4.6.1, and 95% confidence intervals were obtained using 2000 bootstrap resamples. Because these reliability estimates were based on the prespecified subscale scoring structure rather than the cross-loading structure identified in the Target ESEM, they were interpreted as reliability estimates for the proposed summed subscale scores.

For subsequent subgroup and construct-validity analyses, subscale scores were calculated according to the prespecified item allocation to maintain consistency with the original CKD-SE scoring framework. These scores did not incorporate the cross-loadings estimated in the Target ESEM and were therefore treated as proposed summed subscale scores rather than as factor scores derived from the Target ESEM. Exploratory subgroup comparisons were conducted to examine differences in CKDSE-J subscale scores according to sex, age group, and CKD stage. Independent-samples *t*-tests were used for comparisons by sex, and one-way analysis of variance (ANOVA) with Tukey–Kramer post hoc tests was used for comparisons by age group and CKD stage. Before ANOVA, homogeneity of variances was assessed using Levene’s test.

Construct validity was further examined through hypothesis testing by calculating Pearson’s correlation coefficients between CKDSE-J subscale scores and CKDSC-J scores. These analyses were conducted using the 222 participants with complete data on both the CKDSE-J and CKDSC-J. Based on previous research in chronic diseases demonstrating positive associations between disease-specific self-efficacy and self-care behaviors in adults with CKD [5], moderate positive correlations were hypothesized. All statistical tests were two-tailed, and *p* < 0.05 was considered statistically significant.

## 3. Results

Supplementary Figure S1 presents a flow diagram of participant recruitment and analysis-specific sample sizes. A total of 275 eligible patients received questionnaires, including 262 outpatients and 13 inpatients at recruitment. Of these, 244 returned the questionnaires (response rate, 88.7%). Seventeen respondents had missing CKDSE-J item responses, leaving 227 participants with complete CKDSE-J data for the structural validity and internal consistency analyses. Among these participants, five had missing CKDSC-J item responses; consequently, 222 participants with complete data on both the CKDSE-J and CKDSC-J were included in the construct validity analyses involving the CKDSC-J. The demographic and clinical characteristics of the 222 participants included in the construct validity analyses and the five participants with missing CKDSC-J data are presented descriptively in Supplementary Table S2.

### 3.1. Participant Characteristics

Table 1 presents the detailed characteristics of 227 adults with CKD included in the analyses. The mean eGFR at the time of the survey was 33.5 ± 15.8 mL/min/1.73 m^2^. Of the 227 participants, seven had an eGFR ≥ 60 mL/min/1.73 m^2^ at the time of the survey and one had no survey-time eGFR measurement; therefore, CKD stage at the time of the survey was classified for 219 participants. Among the participants, 56.8% were aged 65 years or older, while 33.9% had received a CKD diagnosis within the past one to five years. Education about lifestyle for CKD was provided by healthcare professionals and physicians to 92.5% and 83.7% of participants, respectively.

### 3.2. Item-Level Description of CKDSE-J

Table 2 presents the mean, standard deviation, skewness, and kurtosis for each of the 25 CKDSE-J items. No items exhibited excessive skewness or kurtosis. Mean item scores ranged from 5.0 to 7.8.

### 3.3. Structural Validity

Table 3 summarizes the fit of the structural models. The original 25-item four-factor CFA showed poor overall fit, with χ^2^ (269) = 849.75, *p* < 0.001, CFI = 0.782, TLI = 0.757, RMSEA = 0.098 (90% CI [0.090, 0.105]), and SRMR = 0.089. Diagnostic examination of local model misfit identified particularly large modification indices between Items 22 and 23 (MI = 224.981) and between Items 15 and 19 (MI = 60.861). These item pairs were also strongly correlated (r = 0.903 and 0.745, respectively).

Given the poor overall fit of the 25-item CFA, the original 25-item four-factor structure was subsequently estimated using ESEM with oblique GEOMIN rotation. Model fit improved relative to the CFA, with χ^2^ (206) = 463.74, *p* < 0.001, CFI = 0.903, TLI = 0.859, RMSEA = 0.074 (90% CI [0.065, 0.083]), and SRMR = 0.040. However, this solution was inadmissible because Item 19 had a negative residual variance (−0.339), indicating a Heywood case. Therefore, the 25-item ESEM was not retained as the final structural model.

To determine whether removal of Item 19 alone resolved the measurement problem, the resulting 24-item structure was subsequently evaluated. Although the 24-item ESEM yielded an admissible solution with no negative residual variances, the corresponding 24-item CFA continued to show inadequate model fit, with χ^2^ (246) = 655.94, *p* < 0.001, CFI = 0.832, TLI = 0.811, RMSEA = 0.086 (90% CI [0.078, 0.094]), and SRMR = 0.097. Moreover, substantial local dependence remained between Items 22 and 23 (MI = 142.601), which were strongly correlated (r = 0.903).

The item-removal decisions were then considered in conjunction with item content. Items 15 and 19 both addressed self-efficacy for dietary management in social situations. Item 15 referred to concrete situations such as weddings and banquets, whereas Item 19 referred more generally to social activities. Given this conceptual overlap and the measurement problem involving Item 19 in the 25-item ESEM, Item 15 was retained and Item 19 was excluded. Items 22 and 23 both addressed self-efficacy for dietary management according to recommendations from healthcare professionals. Item 22 focused on adherence to recommended dietary restrictions, whereas Item 23 focused more broadly on adjusting eating habits according to professional advice. Considering the substantial local dependence and conceptual overlap between Items 22 and 23, Item 22 was retained and Item 23 was excluded.

The resulting 23-item structure was then evaluated using both CFA and Target ESEM. The 23-item four-factor CFA showed mixed fit across indices, with χ^2^ (224) = 523.94, *p* < 0.001, CFI = 0.859, TLI = 0.841, RMSEA = 0.077 (90% CI [0.068, 0.085]), and SRMR = 0.068. The standardized CFA solution is presented in Supplementary Figure S2. All prespecified CFA loadings were statistically significant and ranged from 0.477 to 0.853; however, latent factor correlations remained high (0.692–0.902), including correlations of 0.902 between autonomy and problem-solving and 0.855 between autonomy and seeking social support.

**Figure 1:**
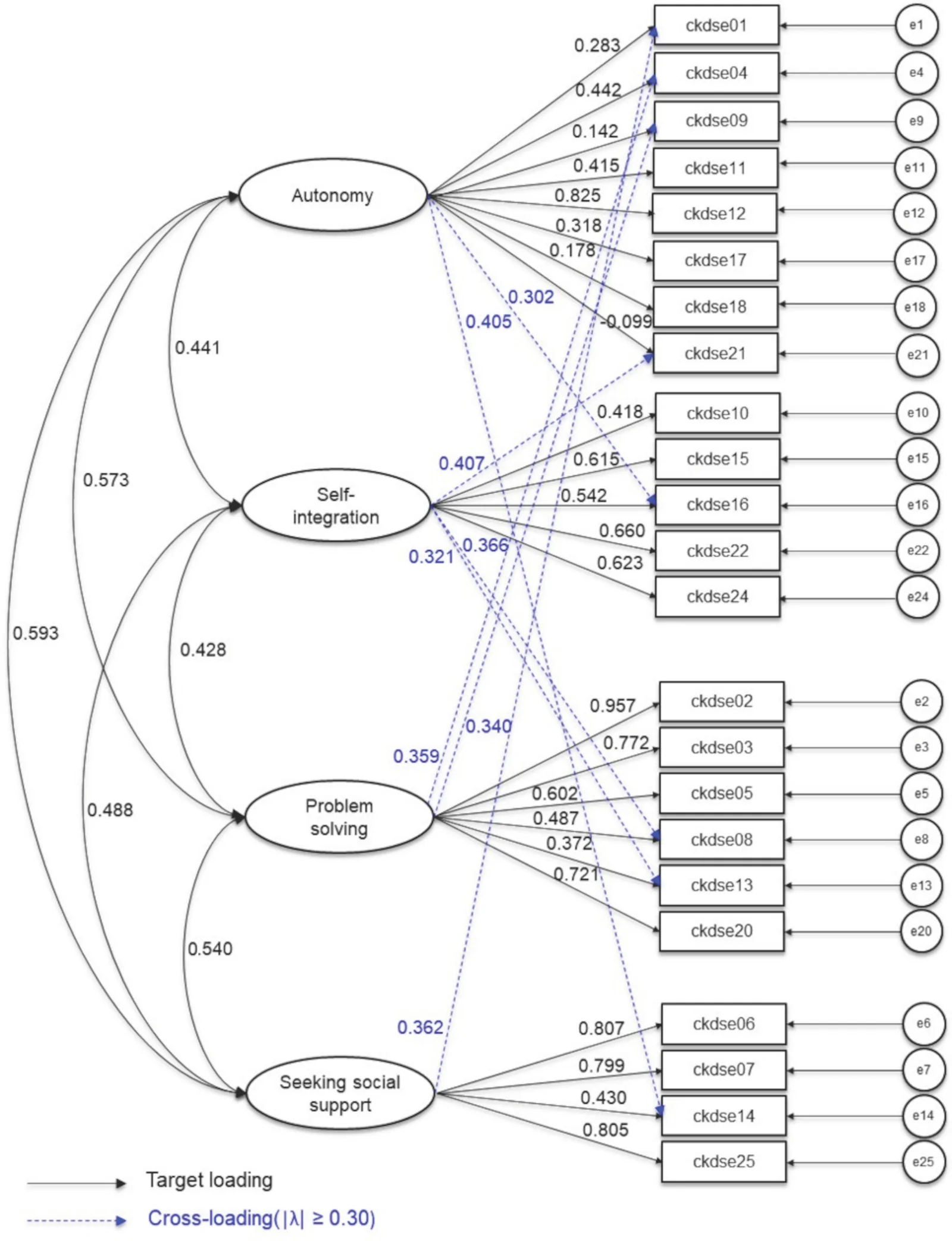
23-item four-factor Target ESEM model of the CKDSE-J. Note. Standardized estimates are shown. Solid arrows represent target loadings, and dashed arrows represent non-target cross-loadings with |λ| ≥ 0.30. Cross-loadings with |λ| < 0.30 are omitted for visual clarity. Curved double-headed arrows represent factor correlations.

The four-factor Target ESEM for the same 23 items showed improved fit, with χ^2^ (167) = 305.16, *p* < 0.001, CFI = 0.935, TLI = 0.902, RMSEA = 0.060 (90% CI [0.050, 0.071]), and SRMR = 0.033. AIC and BIC were also lower for the 23-item Target ESEM than for the corresponding 23-item CFA (Table 3). Factor correlations were lower in the Target ESEM (0.428–0.593) than in the CFA (0.692–0.902). The Target ESEM yielded an admissible solution, with all standardized residual variance point estimates being positive. The standardized Target ESEM solution is presented in Figure 1, and the complete standardized factor-loading matrix, standard errors, residual variances, and factor correlations are presented in Table 4. As shown in Table 4, target loadings were consistently positive for self-integration (0.418–0.660), problem-solving (0.372–0.957), and seeking social support (0.430–0.807). In contrast, the autonomy factor was less clearly defined by its hypothesized items, with target loadings ranging from −0.099 to 0.825. Items 9, 18, and 21 showed weak target loadings (0.142, 0.178, and −0.099, respectively), while Item 17 had a target loading of 0.318. Several autonomy items also showed notable non-target loadings; notably, Item 21 loaded more strongly on self-integration (0.407) than on its hypothesized autonomy factor (−0.099). Thus, although the Target ESEM showed improved overall model fit relative to the corresponding CFA, the autonomy factor remained less clearly differentiated at the item level. A sensitivity analysis restricted to the 219 participants who could be classified as CKD stages G3a–G5 on the basis of survey-time eGFR yielded results comparable to those of the primary analysis (*n* = 227). Model fit, standardized loading patterns, and factor correlations for both the CFA and Target ESEM were essentially unchanged (Supplementary Table S3).

### 3.4. Internal Consistency

Internal consistency was evaluated using the 227 participants with complete CKDSE-J data. McDonald’s omega was 0.84 (95% CI [0.80, 0.87]) for autonomy, 0.84 (95% CI [0.79, 0.87]) for self-integration, 0.90 (95% CI [0.87, 0.92]) for problem-solving, and 0.88 (95% CI [0.84, 0.91]) for seeking social support. The omega coefficient for the total scale was 0.95 (95% CI [0.93, 0.96]). All estimates exceeded the prespecified threshold of 0.70, indicating acceptable internal consistency. For descriptive purposes, sample-specific summary statistics for the proposed subscale scores, calculated according to the prespecified item allocation of the original CKD-SE, are provided in Supplementary Table S4. These descriptive values are not intended as normative values or clinical cutoffs.

### 3.5. Exploratory Subgroup Comparison

Table 5 presents exploratory comparisons of the proposed CKDSE-J subscale scores by sex, age group, and CKD stage. No significant differences were observed by sex or CKD stage. Self-integration scores were significantly higher among participants aged ≥75 years than among those aged <65 years, whereas no significant age-group differences were observed for autonomy, problem-solving, or seeking social support. These exploratory findings should be interpreted cautiously.

### 3.6. Construct Validity: Hypothesis Testing Against Self-Care Behavior

As shown in Table 6, CKDSE-J subscale scores were positively correlated with CKD self-care behavior. Moderate correlations were observed for autonomy (r = 0.36), self-integration (r = 0.46), and problem-solving (r = 0.39), whereas seeking social support showed a weaker correlation (r = 0.27). All correlations were significant (all *p* < 0.01).

## 4. Discussion

This study translated and culturally adapted the CKD-SE into Japanese and provides preliminary psychometric evidence for its use among Japanese adults with CKD stages G3a–G5. The principal structural finding was that the restrictive four-factor CFA showed mixed model fit for the 23-item CKDSE-J, whereas the four-factor Target ESEM provided a better representation of the data. The 23-item CFA yielded CFI = 0.859, TLI = 0.841, RMSEA = 0.077, and SRMR = 0.068, indicating mixed fit across indices, together with high factor correlations. In contrast, the Target ESEM yielded CFI = 0.935, TLI = 0.902, RMSEA = 0.060, and SRMR = 0.033, and factor correlations were substantially lower (0.428–0.593) than those observed in the CFA (0.692–0.902). This pattern is consistent with the rationale for ESEM in multidimensional measures: when substantively plausible cross-loadings are constrained to zero in CFA, shared item variance may be reflected in elevated factor correlations [26,28].

The Target ESEM findings showed lower factor correlations than the CFA when cross-factor associations at the item level were permitted. Accordingly, the very high factor correlations observed in the CFA may have partly reflected the restrictive zero-cross-loading assumption rather than indicating that the four CKDSE-J factors were empirically indistinguishable, as inflated factor correlations are a recognized consequence of constraining non-negligible cross-loadings to zero [28]. However, the improved overall model fit of the Target ESEM should be distinguished from evidence that each proposed subscale adequately measures its intended construct. Nevertheless, the results do not indicate a perfectly simple four-factor structure. Self-integration, problem-solving, and seeking social support showed comparatively coherent target loading patterns, whereas autonomy contained several weak target loadings and non-negligible cross-loadings. In particular, Items 9, 18, and 21 showed weak target loadings on autonomy, and Item 21 loaded more strongly on self-integration than on its hypothesized autonomy factor. The presence of cross-loadings alone does not necessarily invalidate a multidimensional factor structure, because ESEM does not require all non-target loadings to be exactly zero [28]. However, the combination of relatively weak target loadings and non-negligible cross-loadings for several autonomy items suggests that this factor was less clearly differentiated than the other three factors. This pattern may indicate some overlap between autonomy-related self-efficacy and other aspects of CKD self-efficacy in this Japanese sample. Whether this pattern reflects cultural influences, sample-specific characteristics, or properties of the translated items cannot be determined from the present study and requires further investigation. Accordingly, the present findings indicate improved overall model fit for the proposed four-factor representation under Target ESEM, while the structural definition and interpretation of the autonomy subscale remain uncertain and require further evaluation.

The refinement from 25 to 23 items also requires cautious interpretation. In the 25-item ESEM, Item 19 was associated with an inadmissible solution. Removal of Item 19 alone resolved the improper solution in the 24-item ESEM, demonstrating that deletion of Item 23 was not required to obtain an admissible solution. However, the corresponding 24-item CFA continued to show inadequate global fit, and substantial local dependence remained between Items 22 and 23. Thus, the subsequent removal of Item 23 was considered separately on the basis of both the remaining local dependence and item content.

Items 22 and 23 showed substantial statistical and conceptual overlap. Both items addressed dietary self-efficacy in relation to recommendations from healthcare professionals; however, Item 22 focused directly on adherence to recommended dietary restrictions, whereas Item 23 addressed the broader adaptation of eating habits in response to professional advice. Because adherence to recommended dietary restrictions represents a more specifically defined self-management behavior, Item 22 was retained to preserve this aspect of dietary self-efficacy while reducing redundancy between the two items. This decision represents model refinement based on statistical and content considerations and should not be interpreted as evidence that Item 22 is intrinsically superior to Item 23. This approach reflects the principle that decisions regarding item retention or removal during scale refinement should consider conceptual relevance alongside statistical evidence, an approach also applied in recent psychometric work among adults with stage 3–4 CKD [8,32]. Because item removal and evaluation of the resulting 23-item Target ESEM structure were performed in the same sample, the improvement in model fit should be interpreted as model refinement rather than independent confirmation of the revised structure. Accordingly, the present study should be viewed as an initial structural evaluation providing a specific 23-item model for subsequent testing in an independent sample.

The internal consistency of the CKDSE-J subscales was comparable to that reported for the original CKD-SE (Cronbach’s α = 0.843–0.901 across subscales) [13] and findings reported in an Indonesian CKD population [14]. McDonald’s omega estimates indicated acceptable internal consistency for all four proposed subscales. However, these estimates were based on the prespecified item allocation and did not incorporate the cross-loadings identified in the Target ESEM. In particular, although the autonomy subscale showed acceptable internal consistency, several autonomy items had weak target loadings and meaningful cross-loadings. Thus, consistent with the distinction between internal consistency and dimensional structure [28,31], high internal consistency should not be interpreted as evidence that the proposed subscales are structurally distinct or unidimensional.

Exploratory subgroup analyses showed higher proposed self-integration scores among participants aged ≥75 years than among those aged <65 years, whereas no significant differences were observed by sex or CKD stage. Although this age-related pattern may be consistent with the role of accumulated mastery experiences in the development of self-efficacy [11,33], its interpretation requires caution because the present study did not establish the structural distinctness of the proposed subscales. Further studies are needed to determine whether this finding reflects age-related differences in self-integration or other aspects of CKD-related self-efficacy.

CKDSE-J subscale scores showed weak-to-moderate positive correlations with self-care behavior (r = 0.27–0.46). Positive correlations were observed for all four subscales, with moderate associations for autonomy, self-integration, and problem-solving, broadly consistent with the hypothesized magnitude, whereas the association for seeking social support was weaker than anticipated. However, the proposed CKDSE-J subscale scores were themselves substantially intercorrelated, particularly autonomy with problem-solving (r = 0.79) and seeking social support (r = 0.76). Together with the weak target loadings and cross-loadings observed for several autonomy items in the Target ESEM, these findings limit the extent to which the associations with self-care behavior can be interpreted as evidence for the construct validity of four structurally distinct subscales. Because these analyses were based on summed subscale scores calculated according to the prespecified item allocation of the original CKD-SE, rather than factor scores derived from the Target ESEM, they should be regarded as exploratory hypothesis-testing results for the proposed subscale scores. Nevertheless, the positive associations with self-care behavior are consistent with the theoretical role of self-efficacy as a determinant of self-care [5] and provide preliminary evidence regarding the expected relationships of the prespecified CKDSE-J subscale scores with self-care behavior. The weaker association for seeking social support may reflect that this subscale assesses confidence in obtaining help from others rather than confidence in directly performing self-care behaviors. It may also partly reflect cultural patterns reported in previous studies, including reluctance to seek support to avoid burdening others or disrupting interpersonal harmony [34,35,36].

In Japan, previous CKD self-management studies have assessed self-efficacy using general chronic-disease measures [17,18]; however, these measures are not CKD-specific and may not fully capture CKD-related self-management challenges. The CKDSE-J may provide a CKD-specific approach to assessing self-efficacy across autonomy, self-integration, problem-solving, and seeking social support [13]. Such multidimensional assessment may help characterize areas in which adults with CKD have lower confidence and could potentially inform individualized education and self-management support. From a research perspective, the CKDSE-J may also provide a construct-aligned measure for examining CKD-specific self-efficacy in self-management research and intervention studies. However, interpretation of autonomy as a distinct subscale should remain cautious until its structural definition is replicated and clarified in independent samples. More broadly, these potential clinical and research applications require further evaluation, particularly given the structural uncertainty of the autonomy factor and the need to replicate the revised 23-item structure in an independent sample.

A strength of this study is that it provides an initial psychometric evaluation of a culturally adapted CKD-specific self-efficacy measure in Japanese adults with stages G3a–G5 non-dialysis CKD, a clinically relevant population for self-management support. The use of both CFA and Target ESEM enabled a more nuanced evaluation of the hypothesized four-factor structure, identifying improved overall model fit under the Target ESEM as well as specific areas of structural uncertainty.

This study has several limitations. First, the 23-item structure and Target ESEM were evaluated in the same single-center sample; therefore, the improvement in model fit should be interpreted as model refinement rather than independent validation of the revised structure, and the findings may reflect sample-specific characteristics and may have limited generalizability. The sample size was also modest for estimation of a relatively complex ESEM model, further underscoring the need for replication in a larger independent sample. Parameter stability was not formally evaluated using a resampling procedure with factor alignment across rotated ESEM solutions; therefore, the precision and stability of individual target and cross-loading estimates remain to be established, particularly for the autonomy factor. Replication of the proposed 23-item structure in a larger independent sample is needed. Second, although Target ESEM improved global fit and reduced factor correlations, the evidence for the proposed four-factor structure remains preliminary. In particular, the autonomy factor showed several weak target loadings and non-negligible cross-loadings, indicating that its structural definition and interpretation remain uncertain.

Third, cognitive debriefing was conducted with only two adults with CKD, fewer than the 5–8 respondents suggested in translation and cultural adaptation guidance [15]. Although neither participant reported particular difficulties in understanding or responding to the items, feedback was obtained verbally and was not systematically documented or formally analyzed. Moreover, the debriefing primarily focused on whether the translated items were understandable and whether participants experienced difficulty in responding to them. Patient perspectives on item relevance and comprehensiveness, including whether important aspects of CKD self-efficacy were missing, were not systematically evaluated and therefore remain an unresolved aspect of content validity. Further qualitative evaluation in a larger and more diverse sample of Japanese adults across CKD stages G3a–G5 is needed to strengthen evidence for the content validity of the CKDSE-J, particularly for the autonomy domain.

Fourth, although both outpatients and inpatients completed the questionnaire by self-administration, the setting and return procedures differed, which may have influenced response conditions. In addition, test–retest reliability and measurement invariance across demographic and clinical groups were not examined [8,9], and subgroup analyses were exploratory without adjustment for multiple comparisons. Thus, the present study provides initial evidence primarily regarding structural validity, internal consistency, and construct validity through hypothesis testing, rather than a comprehensive evaluation of all measurement properties of the CKDSE-J. Further studies should examine temporal stability, measurement invariance, and independently test the proposed 23-item structure in larger and more diverse samples.

## 5. Conclusions

This study provides an initial psychometric evaluation of the culturally adapted CKDSE-J among adults with CKD stages G3a–G5 not receiving dialysis. The 23-item Target ESEM showed improved overall model fit compared with CFA, but the structural definition of autonomy remained uncertain. Because the 23-item structure was refined and evaluated in the same sample, independent replication and further evaluation of its measurement properties are needed before broader clinical and research application.

## Figures and Tables

**Table 1:** Participant characteristics (*n* = 227).

Variables		*n*	%
Sex			
Male		150	66.1
Female		77	33.9
Age (years)			
<65		98	43.2
≥65		129	56.8
BMI (kg/m^2^)			
M ± SD	23.6 ± 4.1		
eGFR (mL/min/1.73 m^2^)			
M ± SD	33.5 ± 15.8		
CKD stage ^a^ (*n* = 219)			
G3a		67	30.6
G3b		61	27.9
G4		65	29.7
G5		26	11.8
Comorbidities ^b^			
Hypertension		180	79.3
Hyperlipidemia		122	53.7
Hyperuricemia		110	48.5
Diabetes		70	30.8
Heart disease		26	11.5
Duration of CKD (years)			
<1		26	11.5
1–5		77	33.9
5–10		47	20.7
10–15		23	10.1
15–20		14	6.2
≥20		39	17.2
Receipt of CKD education ^c^			
Yes		210	92.5
Educator ^d^			
Physician		190	83.7
Nurse		46	20.3
Nutritionist		131	57.7
Living situation			
Living alone		36	15.9
Living with family		190	83.7
Missing		1	0.4
Employment status			
Employed		111	48.9
Unemployed		56	24.7
Retired		20	8.8
Other		34	15.0
Missing		6	2.6

Note. BMI = body mass index; eGFR = estimated glomerular filtration rate; CKD = chronic kidney disease; M = mean; SD = standard deviation. ^a^ CKD stage was categorized by eGFR values based on the Kidney Disease: Improving Global Outcomes (KDIGO) clinical practice guidelines (45–59 [G3a], 30–44 [G3b], 15–29 [G4], and <15 [G5] mL/min/1.73 m^2^). Eligibility was determined using the most recent eGFR value available at recruitment (<60 mL/min/1.73 m^2^). Seven participants who met this eligibility criterion had an eGFR ≥60 mL/min/1.73 m^2^ at the time of the survey, and eGFR was unavailable at the time of the survey for one participant. These eight participants were retained in the study sample but were not included in the CKD stage classification. ^b^ Comorbidities indicate the presence of multiple diseases per patient in the electronic medical record. ^c^ Experience of receiving education on lifestyle for CKD from medical staff. ^d^ Occupations of medical staff who educated adults.

**Table 2:** Item-level descriptive statistics for the 25-item CKDSE-J (*n* = 227).

Item	M	SD	Skewness	Kurtosis
Factor 1: Autonomy				
ckdse01	6.4	3.1	−0.6	−0.8
ckdse04	7.8	2.5	−1.2	0.9
ckdse09	5.0	2.8	−0.1	−0.8
ckdse11	6.1	2.6	−0.4	−0.3
ckdse12	6.7	2.3	−0.5	0.1
ckdse17	7.3	2.4	−0.8	0.2
ckdse18	7.6	2.1	−1.0	0.7
ckdse21	5.1	3.0	−0.2	−1.0
Factor 2: Self-integration				
ckdse10	5.9	2.6	−0.4	−0.4
ckdse15	5.8	2.7	−0.2	−0.8
ckdse16	6.1	2.4	−0.3	−0.4
ckdse19	5.6	2.8	−0.3	−0.8
ckdse22	5.9	2.4	−0.3	−0.5
ckdse23	6.4	2.4	−0.7	−0.0
ckdse24	5.3	2.7	−0.2	−0.6
Factor 3: Problem-solving				
ckdse02	6.4	2.6	−0.6	−0.4
ckdse03	7.0	2.4	−0.9	0.3
ckdse05	7.8	2.1	−1.1	1.3
ckdse08	6.0	2.4	−0.3	−0.6
ckdse13	5.5	2.5	−0.4	−0.4
ckdse20	6.3	2.9	−0.6	−0.6
Factor 4: Seeking social support				
ckdse06	7.0	2.6	−0.9	0.2
ckdse07	5.8	2.8	−0.3	−0.7
ckdse14	6.6	2.6	−0.6	−0.4
ckdse25	6.1	2.8	−0.5	−0.6

Note. CKDSE-J = Japanese version of the Chronic Kidney Disease Self-Efficacy Scale; M = mean; SD = standard deviation. Item numbers correspond to those in the 25-item English version of the CKD-SE provided by the original developer for the present Japanese adaptation. Each item is rated on a 0–10 scale, with higher scores indicating greater self-efficacy. Item wording is not reproduced.

**Table 3:** Model fit indices for the CFA and ESEM models (*n* = 227).

Model	χ^2^	df	χ^2^/df	CFI	TLI	RMSEA (90% CI)	SRMR	AIC	BIC
25-item four-factor CFA	849.75	269	3.16	0.782	0.757	0.098 (0.090–0.105)	0.089	23,855.15	24,132.57
25-item four-factor ESEM (GEOMIN) ^a^	463.74	206	2.25	0.903	0.859	0.074 (0.065–0.083)	0.040	23,461.70	23,954.90
24-item four-factor CFA	655.94	246	2.67	0.832	0.811	0.086 (0.078–0.094)	0.097	22,834.67	23,101.82
24-item four-factor ESEM (GEOMIN) ^b^	419.362	186	2.25	0.904	0.858	0.074 (0.065–0.084)	0.039	22,539.36	23,012.00
23-item four-factor CFA	523.94	224	2.34	0.859	0.841	0.077 (0.068–0.085)	0.068	22,055.69	22,312.56
23-item four-factor Target ESEM	305.16	167	1.83	0.935	0.902	0.060 (0.050–0.071)	0.033	21,815.25	22,267.34

Note. CFA = confirmatory factor analysis; ESEM = exploratory structural equation modeling; CFI = comparative fit index; TLI = Tucker–Lewis index; RMSEA = root mean square error of approximation; SRMR = standardized root mean square residual; AIC = Akaike Information Criterion; BIC = Bayesian Information Criterion. ^a^ The 25-item four-factor ESEM was estimated using oblique GEOMIN rotation. Although model fit improved relative to the corresponding CFA, the solution was inadmissible because Item 19 had a negative residual variance (−0.339), indicating a Heywood case. ^b^ The 24-item four-factor ESEM was estimated after removing Item 19 and yielded an admissible solution with no negative residual variances. However, the corresponding 24-item CFA continued to show inadequate model fit. In the 24-item CFA, Items 22 and 23 showed substantial local dependence, supporting further examination of their overlapping content. Item 23 was subsequently excluded based on both the statistical evidence of local dependence and consideration of content overlap with Item 22. The 23-item CFA specified the hypothesized independent-clusters structure, whereas the 23-item Target ESEM retained the hypothesized four-factor structure while allowing non-target loadings to be estimated under oblique target rotation.

**Table 4:** Standardized parameter estimates from the 23-item four-factor Target ESEM (STDYX; *n* = 227).

**Panel A. Standardized Factor Loadings**					
**Item**	**Hypothesized Factor**	**Standardized Factor Loadings (SE)**			
		**Autonomy**	**Self-Integration**	**Problem-Solving**	**Seeking Social Support**
ckdse01	Autonomy	0.283 (0.146)	−0.241 (0.082)	0.127 (0.086)	0.362 (0.111)
ckdse04	Autonomy	0.442 (0.112)	−0.22 (0.096)	0.359 (0.107)	0.064 (0.093)
ckdse09	Autonomy	0.142 (0.135)	0.233 (0.105)	0.340 (0.101)	0.114 (0.112)
ckdse11	Autonomy	0.415 (0.184)	0.138 (0.116)	−0.048 (0.105)	0.286 (0.130)
ckdse12	Autonomy	0.825 (0.190)	0.111 (0.146)	0.032 (0.082)	0.036 (0.094)
ckdse17	Autonomy	0.318 (0.164)	0.218 (0.112)	0.236 (0.106)	0.133 (0.115)
ckdse18	Autonomy	0.178 (0.142)	0.225 (0.129)	0.156 (0.100)	0.106 (0.117)
ckdse21	Autonomy	−0.099 (0.114)	0.407 (0.084)	0.260 (0.090)	0.229 (0.088)
ckdse10	Self-integration	0.082 (0.112)	0.418 (0.091)	0.294 (0.084)	0.102 (0.087)
ckdse15	Self-integration	0.063 (0.095)	0.615 (0.122)	0.075 (0.084)	0.002 (0.101)
ckdse16	Self-integration	0.302 (0.087)	0.542 (0.121)	−0.024 (0.091)	0.066 (0.100)
ckdse22	Self-integration	0.076 (0.102)	0.660 (0.084)	0.104 (0.069)	−0.039 (0.079)
ckdse24	Self-integration	−0.132 (0.106)	0.623 (0.106)	0.006 (0.072)	0.248 (0.078)
ckdse02	Problem-solving	−0.060 (0.072)	−0.146 (0.092)	0.957 (0.080)	0.060 (0.056)
ckdse03	Problem-solving	0.222 (0.067)	−0.079 (0.067)	0.772 (0.075)	−0.039 (0.061)
ckdse05	Problem-solving	0.291 (0.129)	−0.079 (0.067)	0.602 (0.109)	−0.112 (0.102)
ckdse08	Problem-solving	0.080 (0.104)	0.366 (0.087)	0.487 (0.091)	0.045 (0.070)
ckdse13	Problem-solving	0.254 (0.106)	0.321 (0.084)	0.372 (0.072)	0.131 (0.079)
ckdse20	Problem-solving	−0.110 (0.075)	0.109 (0.090)	0.721 (0.066)	0.055 (0.070)
ckdse06	Seeking social support	0.058 (0.128)	−0.094 (0.075)	0.051 (0.078)	0.807 (0.115)
ckdse07	Seeking social support	−0.038 (0.086)	−0.064 (0.073)	0.116 (0.057)	0.799 (0.093)
ckdse14	Seeking social support	0.405 (0.095)	0.195 (0.095)	−0.102 (0.071)	0.430 (0.108)
ckdse25	Seeking social support	0.107 (0.073)	0.087 (0.070)	−0.107 (0.074)	0.805 (0.083)
**Panel B. Standardized Residual Variances**					
**Item**	**Residual Variance (SE)**	**Item**	**Residual Variance (SE)**	**Item**	**Residual Variance (SE)**
ckdse01	0.674 (0.069)	ckdse09	0.557 (0.061)	ckdse17	0.474 (0.059)
ckdse02	0.184 (0.055)	ckdse10	0.475 (0.055)	ckdse18	0.721 (0.079)
ckdse03	0.258 (0.058)	ckdse11	0.538 (0.075)	ckdse20	0.446 (0.067)
ckdse04	0.553 (0.077)	ckdse12	0.151 (0.089)	ckdse21	0.550 (0.064)
ckdse05	0.503 (0.098)	ckdse13	0.261 (0.034)	ckdse22	0.468 (0.064)
ckdse06	0.314 (0.075)	ckdse14	0.356 (0.059)	ckdse24	0.489 (0.079)
ckdse07	0.337 (0.073)	ckdse15	0.532 (0.082)	ckdse25	0.259 (0.055)
ckdse08	0.353 (0.047)	ckdse16	0.429 (0.062)		
**Panel C. Factor Correlations**					
**Factor**	**Autonomy**	**Self-Integration**	**Problem-Solving**	**Seeking Social Support**	
Autonomy	1.000				
Self-integration	0.441 (0.100)	1.000			
Problem-solving	0.573 (0.096)	0.428 (0.107)	1.000		
Seeking social support	0.593 (0.116)	0.488 (0.081)	0.540 (0.072)	1.000	

Note. ESEM = exploratory structural equation modeling; SE = standard error; STDYX = standardized estimates using both latent- and observed-variable variances. Values are presented as standardized estimates (SE). All non-target loadings were freely estimated under oblique target rotation and are reported irrespective of magnitude. Absolute loadings ≥ 0.30 were considered substantively notable for interpretation; this threshold was used as an interpretive rather than an item-deletion criterion.

**Table 5:** Comparison of CKDSE-J subscale scores by sex, age group, and CKD stage (*n* = 222).

			Proposed CKDSE-J Subscale Scores			
			Autonomy	Self-Integration	Problem-Solving	Seeking Social Support
Sex	Male (*n* = 147)		51.86 ± 13.72	28.71 ± 9.63	38.35 ± 12.46	25.07 ± 8.82
	Female (*n* = 75)		52.25 ± 14.83	29.52 ± 10.69	40.56 ± 11.05	26.51 ± 9.83
		*t* value	0.19	0.57	1.29	1.10
		*p* value	0.84	0.57	0.20	0.27
Age	<65 (*n* = 96) ^a^		51.24 ± 14.73	26.64 ± 10.54	39.40 ± 12.00	25.56 ± 9.33
	65–74 (*n* = 73) ^b^		52.77 ± 12.02	30.16 ± 8.42	39.70 ± 10.46	25.14 ± 8.97
	≥75 (*n* = 53) ^c^		52.28 ± 15.61	31.60± 10.15	37.74 ± 14.12	26.13 ± 9.18
		*F* value	0.26	5.18	0.46	0.18
		*p* value	0.77	0.006	0.63	0.84
		Post hoc		c > a		
CKD stage (*n* = 214)	G3a (*n* = 60)		51.62 ± 16.46	28.82 ± 10.83	39.73 ± 12.91	24.55 ± 10.66
	G3b (*n* = 60)		52.95 ± 11.39	30.32 ± 8.63	41.33 ± 10.84	27.77 ± 7.85
	G4 (*n* = 61)		49.57 ± 14.43	27.56 ± 9.99	35.84 ± 11.67	23.90 ± 8.89
	G5 (*n* = 33)		54.79 ± 14.25	29.64 ± 10.73	39.61 ± 13.13	26.09 ± 9.17
		*F* value	1.10	0.82	2.25	2.01
		*p* value	0.35	0.48	0.08	0.10

Note. Values are presented as mean ± SD. CKDSE-J = 23-item Japanese version of the Chronic Kidney Disease Self-Efficacy Scale. Independent-samples *t*-tests were used for comparisons by sex. One-way analysis of variance (ANOVA) with Tukey–Kramer post hoc tests was used for comparisons by age group and CKD stage. Superscript letters (a–c) indicate groups used in post hoc comparisons.

**Table 6:** Correlations among the proposed CKDSE-J subscale scores and with the CKDSC-J total score (*n* = 222).

Scale		CKDSE-J Subscales			
		Autonomy	Self-Integration	Problem-Solving	Seeking Social Support
CKDSE-J	Autonomy	-			
	Self-integration	0.66 **	-		
	Problem-solving	0.79 **	0.61 **	-	
	Seeking social support	0.76 **	0.60 **	0.60 **	-
CKDSC-J	CKDSC-J total score	0.36 **	0.46 **	0.39 **	0.27 **

Note. CKDSE-J = Japanese version of the Chronic Kidney Disease Self-Efficacy Scale (23 items); CKDSC-J = Japanese version of the Chronic Kidney Disease Self-care Scale. CKDSE-J subscale scores were calculated according to the prespecified item allocation of the original CKD-SE and should be interpreted as proposed summed subscale scores rather than factor scores derived from the Target ESEM. Pearson’s correlation coefficients were calculated to examine associations between the variables. ** *p* < 0.01.

## Data Availability

The data presented in this study are available from the corresponding author upon reasonable request. The data are not publicly available due to privacy and ethical considerations.

## Supplementary Materials

The following supporting information can be downloaded at https://www.mdpi.com/article/10.3390/healthcare14203393/s1, Figure S1: Flow diagram of participant recruitment and analysis-specific sample sizes; Figure S2: Standardized confirmatory factor analysis solution for the 23-item four-factor CKDSE-J; Table S1: Scoring structure and response options of the proposed 23-item CKDSE-J; Table S2: Comparison of participant characteristics according to CKDSC-J data completeness among participants with complete CKDSE-J data; Table S3: Sensitivity analysis of the 23-item CKDSE-J factor structure; Table S4: Descriptive statistics for the proposed CKDSE-J subscale scores based on the prespecified item allocation in the study sample; Text S1: Mplus syntax for the 23-item four-factor Target ESEM.
