# Supplementary material for: Cross-Cultural Adaptation and Initial Psychometric Evaluation of the Japanese Version of the Chronic Kidney Disease Self-Efficacy Scale (CKDSE-J)
Source: Healthcare (Basel). 2026 Oct 12;14(20):3393. doi: 10.3390/healthcare14203393 (PMC13704021; doi:10.3390/healthcare14203393)
Supplement: Supplementary file 1 [file healthcare-14-03393-s001.zip › healthcare-4544713-supplementary.pdf]

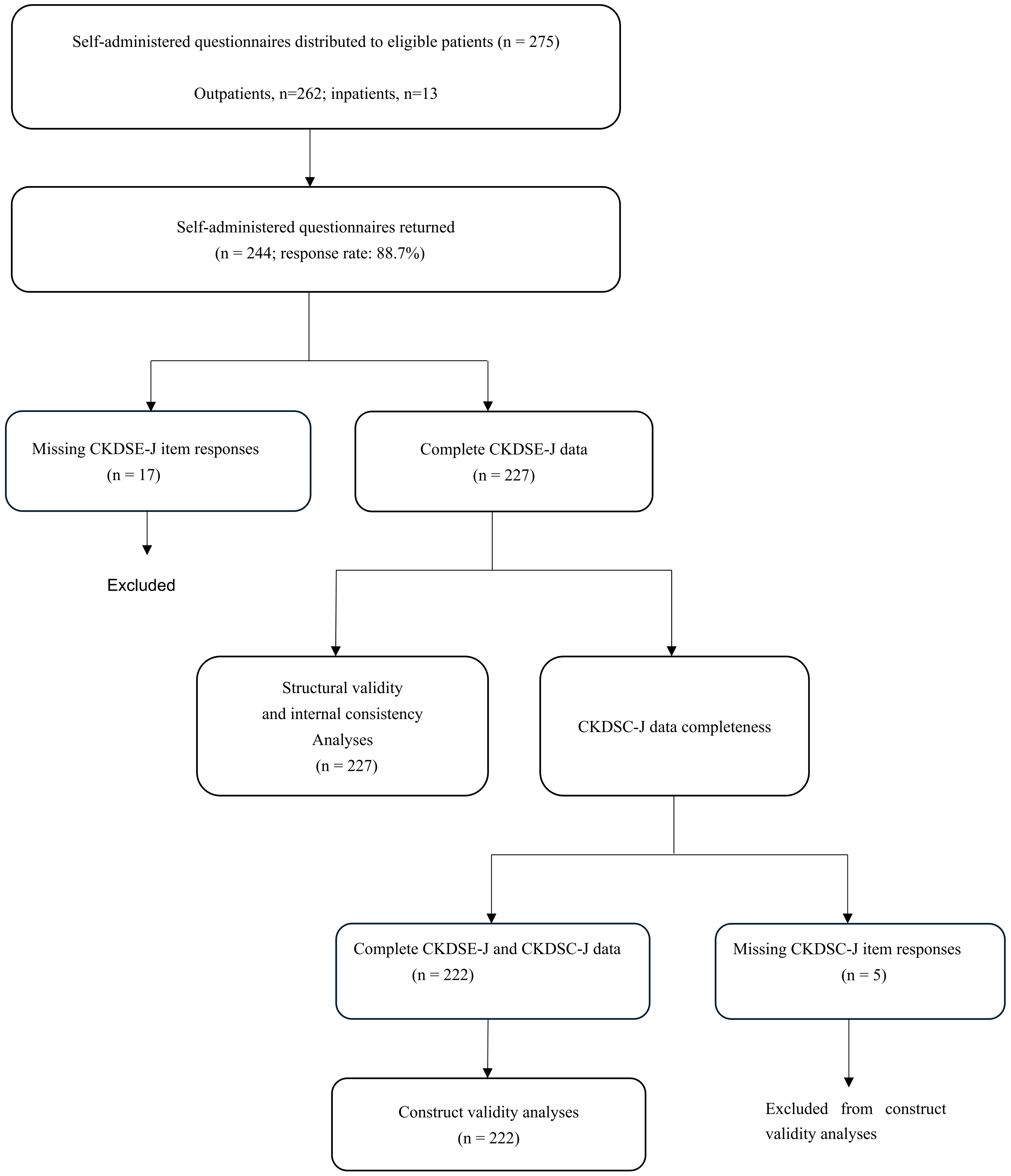

Supplementary Figure S1. Flow diagram of participant recruitment and analysis-specific sample sizes

Note. Of the 275 eligible patients who received questionnaires, 262 were outpatients and 13 were inpatients at recruitment. Recruitment setting was not retained at the individual level in the analytical dataset; therefore, setting-specific response and analysis counts could not be determined. Analysis-specific samples were defined according to the data required for each analysis.

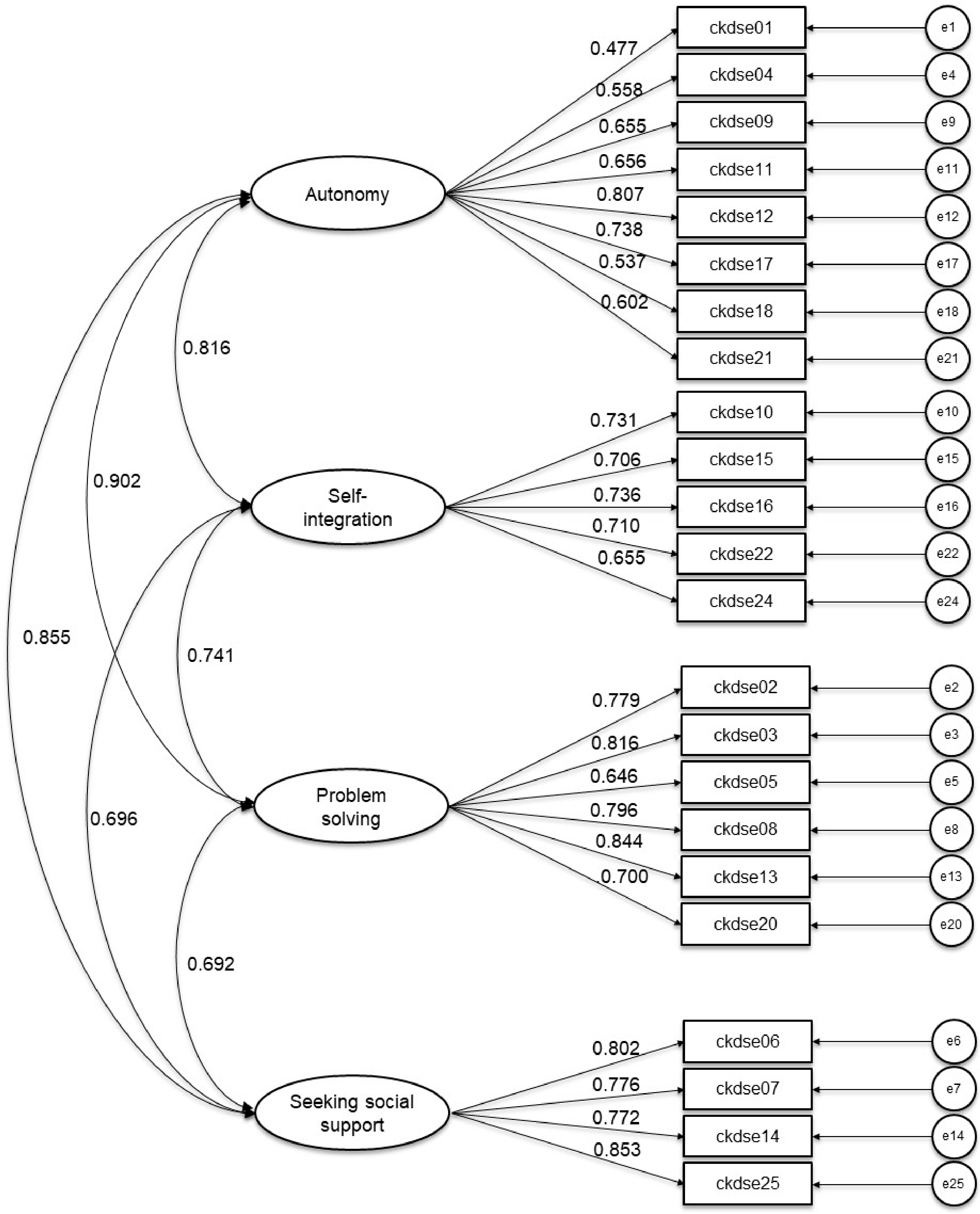

Supplementary Figure S2. 23-item four-factor CFA model of the CKDSE-J

Note. Values represent standardized (STDYX) estimates.

Curved double-headed arrows indicate correlations between factors.

Supplementary Text S1. Mplus syntax for the 23-item four-factor Target ESEM

The following Mplus 7.4 syntax was used to estimate the proposed 23-item four-factor Target ESEM using robust maximum likelihood estimation and oblique target rotation. Items 19 and 23 were excluded from the 23-item model as described in the main text.

```
TITLE: CKDSE 23-item 4-factor Target ESEM;

DATA:
   FILE IS "ckdse227.csv";

VARIABLE:
   NAMES ARE
   id ckdse01 ckdse02 ckdse03 ckdse04 ckdse05 ckdse06 ckdse07
   ckdse08 ckdse09 ckdse10 ckdse11 ckdse12 ckdse13 ckdse14 ckdse15
   ckdse16 ckdse17 ckdse18 ckdse19 ckdse20 ckdse21 ckdse22 ckdse23
   ckdse24 ckdse25;

   USEVARIABLES ARE
   ckdse01 ckdse02 ckdse03 ckdse04 ckdse05
   ckdse06 ckdse07 ckdse08 ckdse09 ckdse10
   ckdse11 ckdse12 ckdse13 ckdse14 ckdse15
   ckdse16 ckdse17 ckdse18 ckdse20 ckdse21
   ckdse22 ckdse24 ckdse25;

ANALYSIS:
   ESTIMATOR = MLR;
   ROTATION = TARGET(OBLIQUE);

MODEL:
   autono BY
   ckdse01 ckdse04 ckdse09 ckdse11 ckdse12 ckdse17 ckdse18 ckdse21
   ckdse02~0 ckdse03~0 ckdse05~0 ckdse06~0 ckdse07~0 ckdse08~0
   ckdse10~0 ckdse13~0 ckdse14~0 ckdse15~0 ckdse16~0 ckdse20~0
   ckdse22~0 ckdse24~0 ckdse25~0 (*1);

   integ BY
   ckdse10 ckdse15 ckdse16 ckdse22 ckdse24
   ckdse01~0 ckdse02~0 ckdse03~0 ckdse04~0 ckdse05~0
   ckdse06~0 ckdse07~0 ckdse08~0 ckdse09~0
   ckdse11~0 ckdse12~0 ckdse13~0 ckdse14~0
   ckdse17~0 ckdse18~0 ckdse20~0 ckdse21~0 ckdse25~0 (*1);

   problem BY
   ckdse02 ckdse03 ckdse05 ckdse08 ckdse13 ckdse20
   ckdse01~0 ckdse04~0 ckdse06~0 ckdse07~0 ckdse09~0
   ckdse10~0 ckdse11~0 ckdse12~0 ckdse14~0 ckdse15~0
   ckdse16~0 ckdse17~0 ckdse18~0 ckdse21~0
   ckdse22~0 ckdse24~0 ckdse25~0 (*1);

   seeking BY
   ckdse06 ckdse07 ckdse14 ckdse25
   ckdse01~0 ckdse02~0 ckdse03~0 ckdse04~0 ckdse05~0
   ckdse08~0 ckdse09~0 ckdse10~0 ckdse11~0 ckdse12~0
   ckdse13~0 ckdse15~0 ckdse16~0 ckdse17~0 ckdse18~0
   ckdse20~0 ckdse21~0 ckdse22~0 ckdse24~0 (*1);

OUTPUT:
   SAMPSTAT;
   STANDARDIZED;
   CINTERVAL;
   TECH4;
```

Supplementary Table S1. Scoring structure and response options of the proposed 23-item CKDSE-J

Proposed subscale	Item numbers	Number of items
Autonomy	1, 4, 9, 11, 12, 17, 18, 21	8
Self-integration	10, 15, 16, 22, 24	5
Problem-solving	2, 3, 5, 8, 13, 20	6
Seeking social support	6, 7, 14, 25	4

Response options:
0 (“no confidence” [全くあてはまらない（全く自信がない）]) to 10 (“great confidence” [とてもあてはまる（とても自信がある）]).

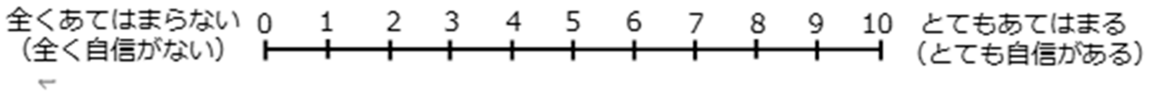

Scoring: Responses are scored directly according to the selected response value (0 = 0 points, 1 = 1 point, …, 10 = 10 points), with higher scores indicating greater CKD-related self-efficacy. Subscale scores are calculated according to the prespecified item allocation shown above.

Note. Items 19 and 23 of the original 25-item version were excluded during scale refinement. The complete Japanese item wording is not reproduced because permission to publicly reproduce the full translated instrument was not included in the authorization obtained from the original instrument developers.

Supplementary Table S2. Comparison of participant characteristics according to CKDSC-J data completeness among participants with complete CKDSE-J data

Variables		Complete CKDSＥ-J and CKDSC-J （n＝222）		Missing CKDSC-J (n=5)	
		n	%	n	%
Sex					
	Male	147	66.2	3	60.0
	Female	75	33.8	2	40.0
Age (years), M ± SD		64.7 ± 12.7		68.6 ± 9.1	
BMI (kg/m²), M ± SD		23.7 ± 4.1		22.0 ± 3.0	
eGFR (mL/min /1.73m²), M ± SD		32.9 ± 15.2		22.3± 6.8	
CKD stage[a]（n=214, n=5）					
	G3a	60	28.0	0	0.0
	G3b	60	28.0	1	20.0
	G4	61	28.5	4	80.0
	G5	33	15.4	0	0.0
Duration of CKD (years) (n=222, n=4)					
	< 1	26	11.7	0	0.0
	1–5	76	34.2	1	25.0
	5–10	47	21.2	0	0.0
	10–15	21	9.5	2	50.0
	15–20	13	5.9	1	25.0
	≥ 20	39	17.6	0	
Receipt of CKD education[b]					
	Yes	205	92.3	5	100.0
Living situation (n=221, n=5)					
	Living alone	35	15.8	1	20.0
	Living with family	186	84.2	4	80.0
Employment status (n=216, n=5)					
	Employed	108	50.0	3	60.0
	Unemployed	55	25.5	1	20.0
	Retired	20	9.3	0	0.0
	Other	33	15.3	1	20.0

Note. BMI = body mass index; eGFR = estimated glomerular filtration rate; M = mean; SD = standard deviation.

[a] CKD stage was categorized according to eGFR values available at the time of the survey, based on the Kidney Disease: Improving Global Outcomes (KDIGO) classification (45–59 [G3a], 30–44 [G3b], 15–29 [G4], and <15 [G5] mL/min/1.73 m²). Eligibility was determined using the most recent eGFR value available at recruitment (<60 mL/min/1.73 m²). Among the 222 participants with complete CKDSE-J and CKDSC-J data, seven had an eGFR ≥60 mL/min/1.73 m² at the time of the survey and one had no eGFR measurement available at that time. These eight participants were retained in the analytical sample but were not included in the CKD stage classification.

[b] Experience of receiving education on lifestyle for CKD from medical staff.

Supplementary Table S3. Sensitivity analysis of the 23-item CKDSE-J factor structure

Panel A. Comparison of model fit between the primary and sensitivity analysis

Model	n	χ^2(df)	CFI	TLI	RMSEA (90%CI)	SRMR
23-item four-factor CFA	227	523.936(224)	0.859	0.841	0.077 (0.068-0.085)	0.068
23-item four-factor Target ESEM	227	305.157(167)	0.935	0.902	0.060 (0.050-0.071)	0.033
23-item four-factor CFA	219	517.723(224)	0.859	0.841	0.077 (0.069-0.086)	0.067
23-item four-factor Target ESEM	219	302.385(167)	0.935	0.902	0.061 (0.050-0.072)	0.034

Panel B. Standardized CFA factor loadings for the autonomy factor

Item	Primary analysis (n = 227)	Sensitivity analysis (n = 219)	Difference (sensitivity - primary)
ckdse01	0.477	0.481	0.004
ckdse04	0.558	0.562	0.004
ckdse09	0.655	0.652	-0.003
ckdse11	0.656	0.659	0.003
ckdse12	0.807	0.813	0.006
ckdse17	0.738	0.740	0.002
ckdse18	0.537	0.546	0.009
ckdse21	0.602	0.605	0.003

Panel C. Comparison of factor correlations in the 23-item four-factor CFA

Factor correlations	Primary analysis (n = 227)	Sensitivity analysis (n = 219)
Autonomy–self-integration	0.816	0.837
Autonomy–Problem solving	0.902	0.902
Autonomy–Seeking social support	0.855	0.862
Self-integration–Problem solving	0.741	0.759
Self-Integration–Seeking social support	0.696	0.690
Problem solving–Seeking social support	0.692	0.701

Panel D. Comparison of factor correlations in the 23-item four-factor Target ESEM

Factor correlations	Primary analysis (n = 227)	Sensitivity analysis (n = 219)
Autonomy–self-integration	0.441	0.434
Autonomy–Problem solving	0.573	0.563
Autonomy–Seeking social support	0.593	0.575
Self-integration–Problem solving	0.428	0.439
Self-Integration–Seeking social support	0.488	0.488
Problem solving–Seeking social support	0.540	0.547

Note. CFA = confirmatory factor analysis; ESEM = exploratory structural equation modeling; CFI = comparative fit index; TLI = Tucker–Lewis index; RMSEA = root mean square error of approximation; SRMR = standardized root mean square residual; STDYX = standardized estimates using both latent- and observed-variable variances.

The primary analysis included 227 participants with complete CKDSE-J data. The sensitivity analysis excluded eight participants who could not be classified as CKD stages G3a–G5 on the basis of survey-time eGFR, resulting in n = 219.

Because the autonomy factor was the principal area of structural concern in the primary analysis, Panel B presents item-level comparisons of standardized CFA loadings for the autonomy factor. In the sensitivity analysis, all standardized CFA loadings for autonomy remained statistically significant and were highly comparable to those in the primary analysis.

Supplementary Table S4. Descriptive statistics for the proposed CKDSE-J subscale scores based on the prespecified item allocation in the study sample (n = 227)

Proposed subscale	No. of items	Subscale score, Mean ± SD	Median	IQR(Q1-Q3)	Min-Max	Item mean score (0–10), Mean ± SD
Autonomy	8	51.9±14.0	52.0	44.0-62.0	7-80	6.5±1.8
Self-integration	5	29.0±9.9	29.0	24.0-35.0	2-50	5.8±2.0
Problem-solving	6	39.1±12.0	42.0	33.0-47.0	8-60	6.5±2.0
Seeking social support	4	25.6±9.2	27.0	20.0-32.0	0-40	6.4±2.3

Note. Subscale scores were calculated according to the prespecified item allocation of the original CKD-SE.
The item mean score was calculated by dividing each subscale score by the number of items in the corresponding subscale and is presented descriptively to facilitate comparison across subscales with different numbers of items. These values represent sample-specific descriptive statistics and should not be interpreted as normative values, clinical cutoffs, or factor scores derived from the Target ESEM.
